# Interdisciplinary Approaches Suggested for Children With Multiple Hospital Referrals Presenting With Non-specific Conditions

**DOI:** 10.3389/fped.2021.656939

**Published:** 2021-04-07

**Authors:** Irene Elgen, Ragnhild Lygre, Gottfried Greve, Silja Griffiths, Torhild Heggestad

**Affiliations:** ^1^Division of Psychiatry, Department of Child and Adolescent Psychiatry, Haukeland University Hospital, Bergen, Norway; ^2^Department of Clinical Medicine, University of Bergen, Bergen, Norway; ^3^Department of Clinical Science, University of Bergen, Bergen, Norway; ^4^Department of Heart Disease, Haukeland University Hospital, Bergen, Norway; ^5^Department of Child and Adolescent Medicine, Haukeland University Hospital, Bergen, Norway; ^6^Department of Research & Development, Haukeland University Hospital, Bergen, Norway

**Keywords:** diagnostic overshadowing, patient flow, mental health, multi-referrals, psychosomatics, child, non-specific condition, health services

## Abstract

**Aims:** To describe the care patterns of patients with repeated referrals to both mental and somatic specialist healthcare, and to study their diagnostic processes.

**Methods:** In a previous register study patients aged 6–12 years referred to Haukeland University Hospital from 2013 to 2015, we found 922 children with at least three referrals including both somatic and mental health services. Of these, more than one in four (250) were randomly selected and observed from their first hospital episode ever and further after inclusion followed during their next three referrals or until July 2017. Data on referral patterns and diagnostics were collected from patient hospital records.

**Results:** Mean number of referrals was 6.5 prior to inclusion and 4.2 in the follow-up period. At the end of the study period 15% of patients still had a non-specific diagnosis. During the follow-up period, more than half of the children were again referred across the border between somatic and mental healthcare.

**Conclusion:** Very complex care patterns were found for these patients, who were repeatedly being referred and “crossing over” between mental and somatic healthcare. This indicates a need for more interdisciplinary-based approaches both within specialist care and between different care levels to broaden the perspective and achieve shorter time lag before reaching a diagnostic conclusion.

**Trial Registration:** Data was obtained from Haukeland university hospital the patient registry system No. 2017/12470. Start of registration was April 1th 2017 and patients included was from 2013 to 15.

## Background

Clinicians often encounter children presenting with symptoms or manifestations that are difficult to relate to a specific diagnosis. These complex patients pose a diagnostic challenge, and many will have multiple referrals to various specialties in secondary healthcare services. The result may be either a delayed diagnosis or often no specific diagnosis at all (1–3). The diagnostic process for these complex cases is both time-consuming and expensive, with soaring utilization of resources in often strained health care systems. Such prolonged diagnostic processes will have negative impact on the child and their parents, and also on society as a whole (4–6).

Children's physical symptoms and complaints are rarely “all-or-nothing” phenomena but usually are part of a continuum of symptoms ranging from barely detectable to severe and readily discernible manifestations. Family doctors may find it difficult to know when and how to act in such cases, and will often need advice from a specialist. This difficulty is compounded by the fact that more than one diagnosis may present with similar symptoms. In children, these symptoms are often less defined. Another related challenge is “diagnostic overshadowing,” which occurs when symptoms of one disorder overshadow those of another. This can, in turn, result in an incomplete (or mis-) understanding of the condition, and hence in an incorrect or a missed diagnosis, or a non-conclusive diagnostic process. This is more often seen in children with chronic disorders; for example, in children with hearing loss and behavioral problems, it is often assumed that their behavior is due to their hearing loss, which means any mental health issues present are not addressed (7, 8).

Several factors contribute to a lengthy diagnostic process in specialist healthcare services. Children with unclear or complex conditions may often be referred to multiple specialties concurrently, or they may have several consecutive referrals within a short period of time. Children with complex problems may also be referred from one department to another if their symptoms are found not to fulfill the criteria for a specific diagnosis, or they may be referred to different sub-specialties successively if the previous referral did not provide satisfactory answers to address the patient's problems. A fragmented diagnostic process rarely brings answers to, or resolve, these children's complex conditions, to such extent that resulting treatment and care may be inappropriate and poorly coordinated (9).

There are no existing comprehensive clinical guidance on how to approach children with compound or non-specific conditions, as well as their families. Effective treatment exists for specific diagnoses as part of complex conditions, but a well-defined approach to address concurrent mental health problems and chronic illnesses is, in general, lacking, although there have been reports of family-centered care or practice that can help patients with multi-morbidity and complex medical complaints (10, 11).

We recently reported on complex care patterns in children with repeated referrals to different medical specialties (1). Interdisciplinary care patterns were particularly common for patients referred to mental healthcare. Patients with combined referrals to both somatic and mental healthcare were found to have a higher number of referrals as well as a higher number of different diagnoses. Overall, there was a high frequency of non-specific diagnoses.

The previous study was based on a hospital register of children aged 6–12 years. To learn more about the specified patient group and supplement the earlier methodical approach, we decided to perform a study on a selection of patients with repeated combined referrals.

The aim in the present study was to describe: (1) the care patterns of patients with repeated referrals to both mental and somatic specialist healthcare, (2) their diagnostic processes.

## Methods

The present study was based on a randomly selection of patients from a retrospective hospital register study of complex care patterns (1). The Haukeland University Hospital is a regional hospital providing care across a wide range of clinical specialties, and covering a local catchment area of half a million inhabitants. In the area, only this hospital has specialist healthcare for children. Access to publicly funded specialist services is restricted in Norway with family doctors acting as “gate-keepers.”

The previous register study included patients aged 6–12 years who had at least one hospital episode from January 1, 2013 to December 31, 2015 (1). For the present study we randomly selected a group of patients from the register study for more detailed analyses of the referral patterns and diagnostic processes, and to enable a longer observation period.

### Population

**Figure 1:**
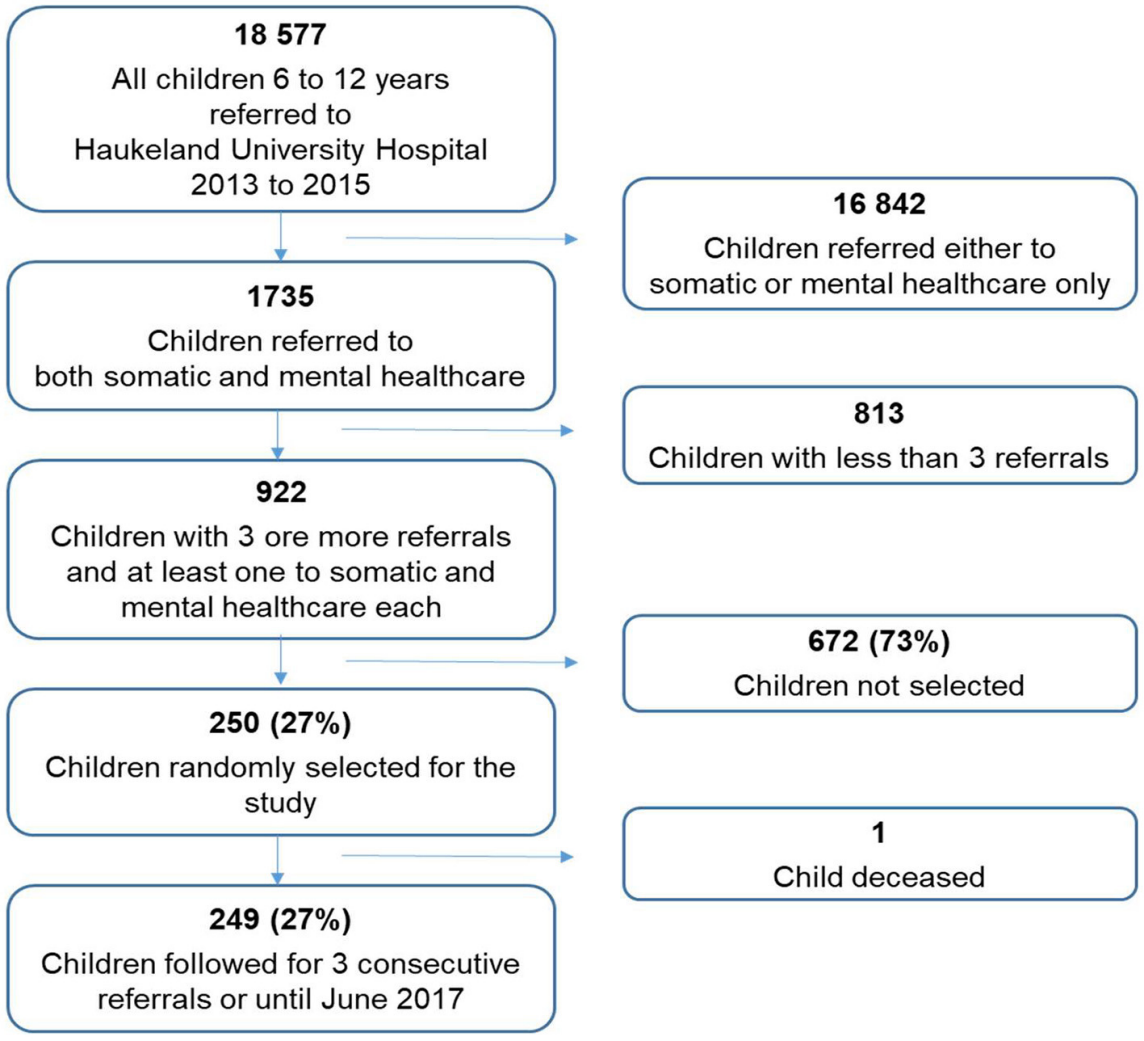
Flow chart showing the selection of study population from the register data of children aged 6–12 years referred to a regional university hospital over a 3-year period (2013–2015).

In this study, we wanted to include patients with a higher probability of having complex care pathways (1). Inclusion criteria or the group of interest (population) was specified as patients with the combination of three or more primary referrals during the 3 year period of inclusion (2013–2015) and with referrals to both somatic and mental healthcare (Figure 1).

The number of patients randomly included in this study population was an estimate of one in four of the total population (922 see Figure 1) which was 250. Power calculation was not feasible since we had no evidence of number of children with complex conditions.

**Figure 2:**
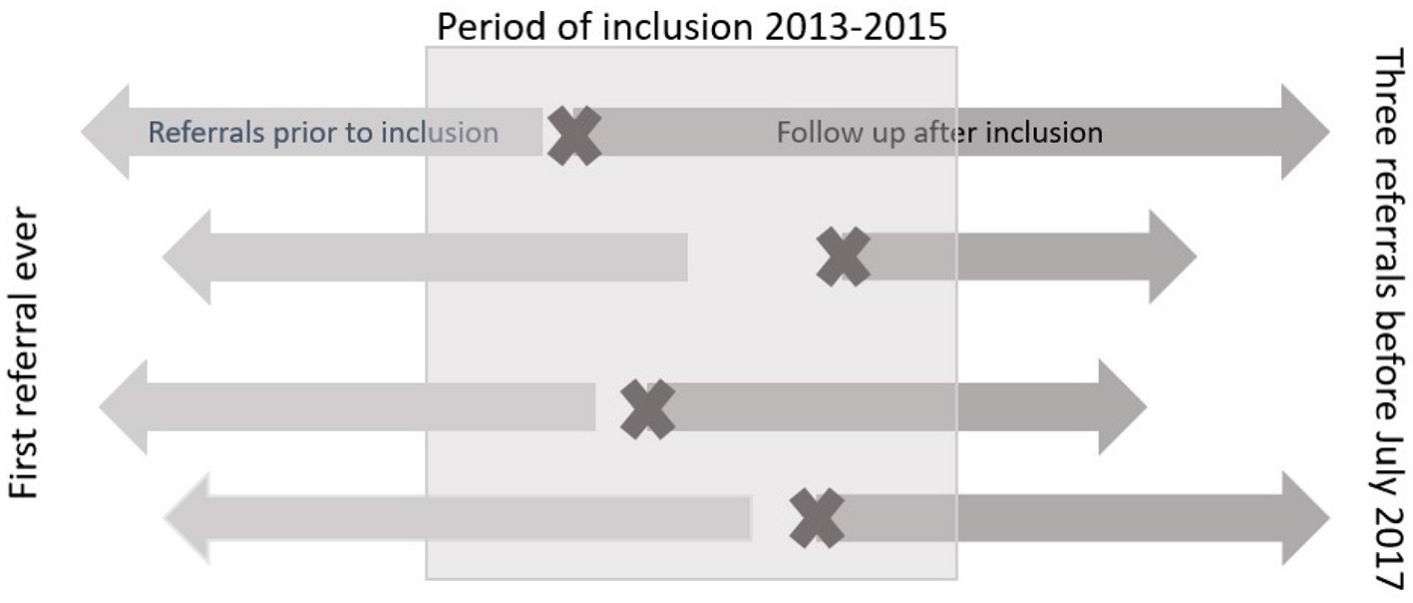
Figure illustrating the design of the different study periods. The pre-inclusion period included all hospital referrals from the first ever until inclusion in the study. Follow-up was continued for three referrals after inclusion or until July 1st 2017. The sign X refers to the referral that merited inclusion i.e., third or more referral including both mental and somatic care service.

We extracted data that included all hospital referrals from their first registered hospital care episode (Figure 2). Data on gender, age, referral patterns, and diagnostic status were collected from the medical records.

### Population in the Observational Period

The observational period started at the first care episode associated with the referral meriting the inclusion (Figure 2). For each patient, data were followed from the time of inclusion through the original time window 2013–2015 and throughout a prolonged observation of hospital care until June 30, 2017 or through the next three referrals from inclusion. Of 250 randomly selected patients, one died and 249 were followed up.

### Referral Patterns

In Norway, every patient referral to specialist healthcare is evaluated by a physician or psychologist according to the Patients' Rights Act. However, patients may be denied access if it is considered to be no indication for further examinations or treatment in the specialist health services. If the patient is granted access, he or she is given priority to care according to (i) disease severity (prognosis as affected by life expectancy and quality of life), (ii) expected effect of available health care, and iii) the cost-effectiveness of the services. All patients given access to care are also assigned a due date within which care has to be initiated.

We collected data on all primary referrals (including both accepted and rejected) both prior to inclusion and in the follow-up period. Further, we collected data on secondary referrals (defined as generated within the hospital) and on the medical specialty of the hospital department receiving the primary referrals.

### Diagnostic Processes

From the medical records, we collected data on the diagnostic status at the last hospital episode related to the studied referral. A senior doctor (IBE) classified the diagnoses as follows: one or more specific diagnoses; no definitive diagnosis; or non-specific condition defined as either R or Z diagnosis according to the International Classification of Diseases, tenth revision (ICD-10) (12). For the group of specific diagnoses in the follow-up period they were further classified as *independent*; having several diagnoses that were not related, or *related* defined as having several diagnoses that were related to the same condition/symptoms.

### Statistical Analyses

A *post hoc* analysis into the margin of error showed that, out of a population of *n* = 922 and a 95% confidence interval, a selection of 250 patients results in a margin error of 5%. For descriptive analyses, we used mean scores and standard deviation (SD). When appropriate groups were compared using *T*-test for means or χ^2^-tests. A *p*-value of < 0.05 was considered statistically significant. The SPSS statistical package version 24.0 (IBM Corporation, Armonk, NY, USA) was used for all analyses (13).

## Results

### Study Population at Inclusion

The selected group of 250 children had a mean age of 82 months (SD: 34 months) at their first referral in the inclusion period, and 66% were boys (Table 1). Age and gender were not significantly different from the remaining group of 672 patients who were not selected into the study.

**Table 1:** Referral patterns and diagnostic status of the 250 patients in the period prior to inclusion in the study^a^.

**At the first referral in the inclusion period**	
Boys	165 (66%)
Mean age in months	82 (SD:34)
**Previous referral patterns (prior to inclusion**^**b**^**)**	
Mean number of all previous primary referrals	6.5 (SD:2.8)
Mean number of previous rejected primary referrals	2.0 (SD:0.4)
Mean number of previous secondary referrals^c^	1.7 (SD:1.6)
**Previous diagnostics (prior to inclusion**^**b**^**)**	
Patients with specific diagnoses	73 (29%)
Patients with no registered diagnoses	129 (52%)
Patients with non-specific diagnoses^d^	48 (19%)

a *Children aged 6–12 years who had at least three referrals including both mental and somatic healthcare to a regional university hospital from 2013 to 2015*.

b *Diagnose from the last episode associated with the last referral before the inclusion period*.

c *Secondary referrals are defined as referrals generated within the hospital*.

d *Non-specific diagnose defined as R or Z chapter diagnoses in the International Classification of Diseases, tenth revision*.

### Referral Patterns From the First Registered Hospital Care Episode

Mean number of primary referrals to the hospital prior to inclusion are presented in Table 1 as well as internal referrals to other specialties within the hospital (i.e., secondary referrals). Referrals for electroencephalography or to the radiology department were not considered.

Of the rejected referrals, 40% were explained by the child suffering from a diagnosed chronic illness where the referral symptoms were explained by the chronic illness. We interpreted this as possible diagnostic overshadowing.

Seventy-seven per cent (*n* = 193) of first referrals ever were made either to child and adolescents mental health services (42%, *n* = 105/250) or to department of pediatrics (35%, *n* = 88/250). The most frequent sub-specialties in pediatrics were gastroenterology and neurology. The remaining 23% (*n* = 57/250) of referrals were most frequently to the specialties of eye, ear, nose, and throat and surgical departments.

### Diagnostics From the First Registered Hospital Care Episode Until Observational Period

The majority of children first referred to child and adolescents mental health services (54%, *n* = 57/105) had symptoms of ADHD, while gastrointestinal symptoms were the most common presentation among children first referred to the pediatric department (32%, *n* = 28/88). For the group of children with ADHD symptoms, 74% (*n* = 42/57) were boys, compared to 68% (*n* = 19/28) with gastrointestinal complaints (OR: 1.1; 95% CI 0.8 to 1.5; *p* = 0.34), and the mean age for the two groups was similar.

By the end of the last referral prior to the inclusion period, almost one in five children had a non-specific diagnose defined as R or Z diagnosis according to the ICD-10 classification (Table 1). Further, almost half of the study population were not given a diagnosis at all. In addition, in the medical records we often found reports of specific diagnoses that were excluded, but less often mentions of other tentative diagnoses (or differentials).

### Referral Patterns in the Observational Period

Mean total number of primary referrals in the follow-up period was 4.2 (SD: 0.8) (Table 2). Most patients had three referrals in the period, whereas 61 (24%) had only two referrals and all of these patients were given specific diagnoses.

**Table 2:** Referral patterns and diagnostic status of the patients in the observational period^a^.

**Referral patterns in the follow-up period**	
Mean total number of primary referrals	4.2 (SD:0.8)
Mean number of rejected primary referrals	0.6 (SD:0.2)
Mean number of secondary referrals^b^	0.6 (SD:0.4)
**Diagnostics in the follow-up period**^**c**^	
Patients with specific diagnoses	213 (85%)
Independent^d^	185
Related^e^	28
Patients with non-specific diagnoses^f^	36 (15%)

a *Characteristics of 250 randomly selected children aged 6–12 years who had at least three referrals including both mental and somatic healthcare to a regional university hospital from 2013 to 2015*.

b *Secondary referrals are defined as referrals generated within the hospital*.

c *Diagnostic data from medical records of the referral by the time of inclusion and those in the follow-up period. The follow-up period included data until June 30, 2017 or two new referrals*.

d *Independent was defined as having several diagnoses that are not related*.

e *Related was defined as having several diagnoses that are related to one another*.

f *Non-specific diagnose defined as either R or Z chapter diagnoses in the International Classification of Diseases, tenth revision*.

Of the 249 patients who were followed up, 167 (67%) were referred to at least two different specialties. For the remaining 82 patients with a repeated single-specialty referral, the most common specialties were: mental health (16%, *n* = 13/82); pediatric department, gastroenterology (11%, *n* = 9/82); and pediatric department, neurology (11%, *n* = 9/82). Furthermore, during the follow-up period a high number of children again were referred across the border between the somatic and mental health services. For the 188 (76%) patients with three referrals the “crossover” patterns are given in Table 3. More than three of four (78%) of patients first referred to mental health services were again referred to somatic health services. Further, 42% of the children starting with a referral to somatic care were again referred to mental health services.

**Table 3:** The care pathway in specialist health services for 249 children^a^.

Inclusion referral	*n*	At 3rd referral	*n*	% of subgroup
Mental care	60			
		Cross over to somatic care	47	78%
		*Still at mental care*	13	
Paediatric dept.	69			
		Cross over to mental care	27	39%
		*Still at somatic care*	42	
Other somatic dept.	59			
		Cross over to mental care	27	45%
		*Still at somatic care*	32	
All patients	188			
		Patients with cross over	101	54%

a *Children (6–12 years of age) with two or more referrals in the inclusion period (2013–2015) and followed for their possible next three referrals or to July 2017*.

Of note, referrals of children with a chronic somatic illness to mental health services were more often rejected, compared to those of children without a chronic illness. Conversely, referrals of children with mental health problems to pediatric medical specialties were more often rejected, compared to those of children without mental health disorders. This was also interpreted as possible diagnostic overshadowing.

### Diagnostics in the Observational Period

The patients' diagnostic status was evaluated and classified at the end of the last referral in the follow-up period (Table 2). All patients had at the end point received a registered diagnosis. For 36 children (15%) they were still found to have a non-specific diagnosis. Studying the diagnostic processes for these patients from the first care episode ever until the end of the observation period, we classified them into three groups. First, 25 of these children had either no diagnoses or non-specific diagnoses during the whole period. These children were, in addition to mental care, twice referred to different sections at the pediatric department, and our interpretation of their symptoms was that these children suffered from psychosomatic conditions. The second category of eight children had specific diagnoses in the follow-up period but they did not receive a final conclusion. All these children had different developmental disturbances. In the last group, three children had independent diagnoses in the pre-inclusion period but received a non-specific diagnose at mental health service in the follow-up period, where two had a syndrome and one fatigue after chemotherapy.

In addition, patients (36) with a non-specific diagnose were referred to an in-house physiotherapist, as part of the diagnostic assessment, compared to 10 (5%) of the remaining 213 children (OR: 3.4; 95% CI 1.3 to 3.9). We found no statistically significant differences in terms of gender, mean age, number of referrals or number of rejected referrals between the “non-specific diagnose” group of 36 children and the other group of 213 children.

## Discussion

In the present study of the care patterns of patients (6 to 12 years) with three or more referrals including both mental and somatic medical specialties, we identified a high number of repeated referrals including rejected ones. During the observational period after inclusion, a high number of children again were referred across the border between the somatic and mental health services. Even at the end of the period, almost one in seven patients still had a non-specific diagnosis. Such patterns taken together is a strong indication of the complexity of these patients and their problems.

The described care patterns rise some questions about the diagnostic processes. One interpretation of the repeated patterns including sequences to different medical specialties, may be that the conditions definitely represented cases where it was difficult to achieve a diagnostic conclusion. However, one may question if not the strength of the symptoms or functional consequences have to be considerable to merit such a high number of repeated referrals. Alternatively, the conclusion of the diagnostic process may have been poorly communicated so that health worries persisted. Anyhow, one may worry about the time lag before reaching a conclusion that were clearly communicated and accepted.

On collection of data representing the diagnostic process we found that while the medical records of children without a specific diagnosis often mentioned excluded specific ones, they rarely mentioned possible differentials. This could be due to limited time availability in a busy clinical practice, but also highly likely due to the uncertainty of which diagnosis to record in these complex cases. The findings also illustrate an earlier registration practice where it was acceptable to leave no registered diagnose. The routine of to-day is to register a non-specific symptom diagnosis if no specific conclusion is reached.

All patients with a non-specific diagnosis at the end of the observation period had a sequential referral pattern that included one to a physiotherapist, even though their symptoms were not primarily motor-related and hence would not normally be addressed and resolved through physiotherapy. Such a situation would prove frustrating to the patient, as well as to their family and physiotherapist, and, in turn, would often lead to further referrals.

We found that referrals of young children to specialist healthcare services were commonly rejected. This could be due to inadequate information in the referrals or that the health-related complaints were deemed inappropriate to be assessed by specialists. Access to specialist assessment has become a challenge in the current healthcare system in terms of prioritizing and organizing health care provision. Rejection of referrals by specialist services could also be a result of diagnostic overshadowing. This interpretation is supported by reports that referrals of children with chronic somatic illnesses are rejected by mental health services more commonly than those of children without chronic illnesses; and conversely referrals of children with mental health problems are rejected by pediatric specialties more often than those of children without.

Huffman et al. described in a recent review that collaborative care interventions including both mental and somatic health care, improve health outcomes and reduce health care costs (14). The fragmentation found in the activity based funding system of services is a main challenge faced within the Norwegian specialist healthcare. Each consultation by a single physician or sequence of such contacts is more highly compensated than are simultaneous consultations by interdisciplinary teams. Accordingly, there is no financial incentives for an interdisciplinary approach to patient care.

For optimal effectiveness, such interdisciplinary approach requires teamwork training, and not mere “collaboration” among colleagues from different specialties. In addition, to implementing the use of interdisciplinary teams, health care professionals should also integrate the concept of shared decision-making (15–17) which involves both the patient and their parents. Such a strategy should not only include treatment/follow-up decisions, but also include choosing of diagnostic strategies (14).

The strengths of the present study are the length of the study period including the first-ever specialist care episode as well as the detailed study of medical records. Weaknesses, however, are the retrospective design and lack of statistical power calculation that was not able to feasible and not including error of margin of error initially. However, the nature of the study was more descriptive and explorative.

Given the trend of multiple referrals starting at a young age in children, we propose that hospital register systems should be set up in a way that alert clinicians of complex cases, with a view to extending the traditional single-disease approach to the use of a biopsychosocial model. Such repeated referrals, often with non-specific conditions, seem particularly challenging to address for an individual specialty.

## Conclusion

Following children 6 to 12 years of age with multi-referrals to different specialist healthcare over an extended time-period from their first care episode ever, we found a high frequency of diagnostic processes ending without a specific conclusion. The findings call for an interdisciplinary-based approach to broaden the perspective and shorten the time lag before reaching a diagnostic conclusion.

## Data Availability Statement

The original contributions presented in the study are included in the article/supplementary material, further inquiries can be directed to the corresponding author/s.

## Ethics Statement

The project was approved by the Data Protection Officer at Haukeland University Hospital (No. 2017/12470). Written informed consent from the participants' legal guardian/next of kin was not required to participate in this study in accordance with the national legislation and the institutional requirements.

## Author Contributions

TH extracted records from the hospital registry. IE and RL collected date from the randomly extracted records of 250 patients analyzed the data. SG and GG were major contributors in writing in addition to the other authors. All authors read and approved the final manuscript.

## Conflict of Interest

The authors declare that the research was conducted in the absence of any commercial or financial relationships that could be construed as a potential conflict of interest.

## Abbreviations

ADHD: Attention deficit hyperactivity disorder
ANOVA: analysis of variance
ICD-10: the International Classification of Diseases tenth revision
SD: Standard deviation
SPSS: statistical package.
